# Use of Patterned Collagen Coated Slides to Study Normal and Scleroderma Lung Fibroblast Migration

**DOI:** 10.1038/s41598-017-02621-3

**Published:** 2017-06-01

**Authors:** Bahja Ahmed Abdi, Henry Lopez, Sarah Karrar, Elisabetta Renzoni, Athol Wells, Angela Tam, Oseme Etomi, J. Justin Hsuan, George R. Martin, Xu Shiwen, Christopher P. Denton, David Abraham, Richard Stratton

**Affiliations:** 1 Centre for Rheumatology and Connective Tissue Disease, Royal Free Hospital Campus, University College Medical School, Rowland Hill Street, London, NW3 2PF UK; 2 MuriGenics, Inc., 941 Railroad Avenue, Vallejo, CA 94592 USA; 3 Imperial College London, Royal Brompton Campus, Sydney Street, London, SW3 6NP UK; 4 Institute for Liver and Digestive Health, Royal Free Hospital Campus, University College Medical School Rowland Hill Street, London, NW3 2PF UK

## Abstract

Systemic sclerosis (SSc) is a spreading fibrotic disease affecting the skin and internal organs. We aimed to model pathogenic fibroblast migration in SSc in order to identify enhancing factors, measure the effect of migrating cells on underlying extracellular matrix (ECM) and test possible therapeutic inhibitors. Novel patterned collagen substrates were used to investigate alignment and migration of skin and lung fibroblasts from SSc patients and healthy controls. Normal lung but not skin fibroblasts consistently elongated and aligned with underlying collagen and migrated dependent on PDGF or serum. SSc lung fibroblasts remained growth factor dependent, did not migrate more rapidly and were less restricted to alignment of the collagen. Multiple collagen proline and lysine-modifying enzymes were identified in SSc but not control fibroblast extracellular matrix preparations, indicating differential levels of ECM modification by the diseased cells. Profiling of migrating cells revealed a possible SCF/c-Kit paracrine mechanism contributing to migration via a subpopulation of cells. Heparin, which binds ligands including PDGF and SCF, and imatininib which blocks downstream tyrosine kinase receptors, both inhibited lung fibroblast migration individually but showed synergy in SSc cells. Pathologic lung fibroblasts from SSc patients modify ECM during migration but remain growth factor dependent and sensitive to inhibitors.

## Introduction

Systemic sclerosis (SSc, scleroderma) is a severe fibrotic disease in which autoimmunity, inflammation, and vascular damage lead to progressively spreading fibrosis of the skin and internal organs, most notably the lung^1, 2^. In SSc, progressive lung fibrosis is the leading cause of mortality^3^. In the earliest stages of SSc, fibroblast activation is initiated in areas of endothelial cell damage in the peripheral dermis, spreading to become generalised and to involve internal organs^4^. Fibrotic changes in the lungs are initially localized to posterior subpleural areas of the lower lobes, which may then extend throughout the lungs over months to years^2, 5^. Cellular migration is likely to have a role in the local progression of fibrosis, permitting recruitment of cells into the fibrotic activated areas and invasion of pathogenic cells into healthy tissue.

The most frequent pathologic pattern in SSc pulmonary disease is non-specific interstitial pneumonitis (NSIP) in which lymphocytic infiltration and inflammatory changes variably accompany extensive fibrotic remodelling^5^. Immunosuppressive therapeutic regimens which combine corticosteroid with cyclophosphamide slow progression of pulmonary involvement in SSc but are associated with an increased risk of sepsis and other adverse effects^6^. High dose mycophenolate mofetil is equivalent in efficacy to the cyclophosphamide regimens with less toxicity^7^. More specific therapies currently under evaluation include tyrosine kinase inhibitors such as imatinib, found to benefit mouse models of fibrosis and to attenuate the progression of lung involvement in one open trial in SSc^8, 9^, and nintedanib, shown to slow disease progression in idiopathic pulmonary fibrosis^10, 11^.

In fibrosis, activated myofibroblasts originate from a number of sources including resident fibroblasts, epithelial cells undergoing epithelial to mesenchymal transition (EMT), perivascular cells and blood derived monocytes (fibrocytes)^12^. A further role for cell migration in the pathogenesis of SSc is in the recruitment of these precursor cells into the fibrotic lesion. In keeping with this, we have found that platelet derived growth factor (PDGF), a known chemoattractant for fibroblasts, is over-expressed in the disease microenvironment in SSc^13^. A better understanding of the mechanisms underlying fibroblast migration might identify targets that could be inhibited in order to block the recruitment of cells into fibrotic lesions. The migration of cells has a role in other important human pathology such as other forms of fibrosis, and cancer stroma invasion^14^.

In order to assess the migration of fibroblasts, slides coated with patterned collagen fibres were used to model the extracellular matrix. They included a woven randomly aligned pattern to model uninjured extracellular matrix and an aligned pattern modelling scar tissue.

The migration of control and SSc derived lung and skin fibroblasts and their responses to candidate factors involved in enhancing migration were studied. Broad screening methods were used to investigate possible modification of the underlying matrix by proteins secreted by migrating cells, and to profile the phosphorylation changes.

## Results

### Control lung fibroblasts align and migrate on aligned collagen fibers

**Figure 1:**
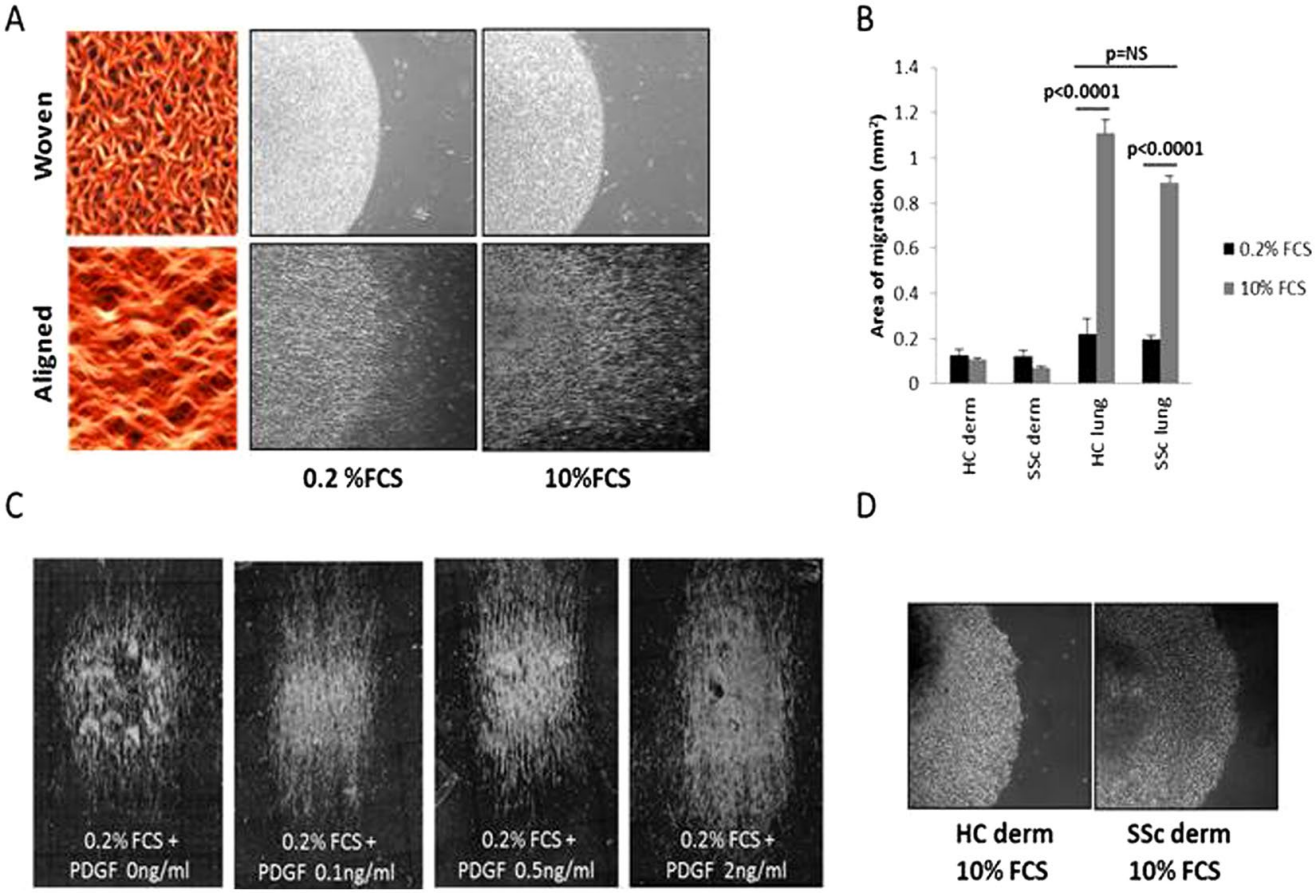
Migration of lung fibroblasts on aligned collagen dependent on serum and PDGF. Fibroblasts were applied to the patterned collagen substrates as 3 µl drops containing 4000 cells per µl, allowed to adhere and then cultured further in DMEM with 0.2% or 10% FCS or with PDGF-BB. Healthy control (HC) lung fibroblasts did not migrate on the woven collagen substrates, but remained as adherent cellular foci. However, when cultured on aligned collagen these cells elongated and migrated parallel to the aligned collagen fibrils, enhanced by 10% FCS. 24 hour time point shown (**A**). HC and SSc lung fibroblasts showed a similar magnitude of enhanced migration with FCS 10% versus 0.2% at 24 hours (**B**). The additon of PDGF 0.1–2.0 ng/ml enhanced normal lung fibroblast migration when cultured on the aligned collagen in media containing FCS 0.2% (**C**). Dermal fibroblasts (derm) did not consistently align or migrate on either substrate, but remained as non-migrating cellular foci, 24 hour time point shown (**D**).

The initial focus was on culturing dermal and lung fibroblasts from healthy controls on aligned or dermis-like (woven pattern) collagen substrates. Aligned collagen fibers are more highly ordered than dermis-like collagen. The collagen fibrils appear visibly directional, in a helix-like, wavy manner. The woven collagen on the other hand is decidedly non-directional with a random appearance (Fig. 1A). Using these collagen substrates, healthy control lung fibroblasts showed aligned linear migration on the aligned pattern collagen matrices but not on the woven substrate. Migration of the lung cells was dependent on the presence of fetal calf serum (FCS) (Fig. 1A,B), or PDGF-BB (0.1–2 ng/ml) (Fig. 1C). In contrast to lung cells, dermal fibroblasts remained in a non-migrating cellular focus both on woven and aligned collagen coated slides and did not consistently align or migrate in response to FCS (Fig. 1B and D).

**Figure 2:**
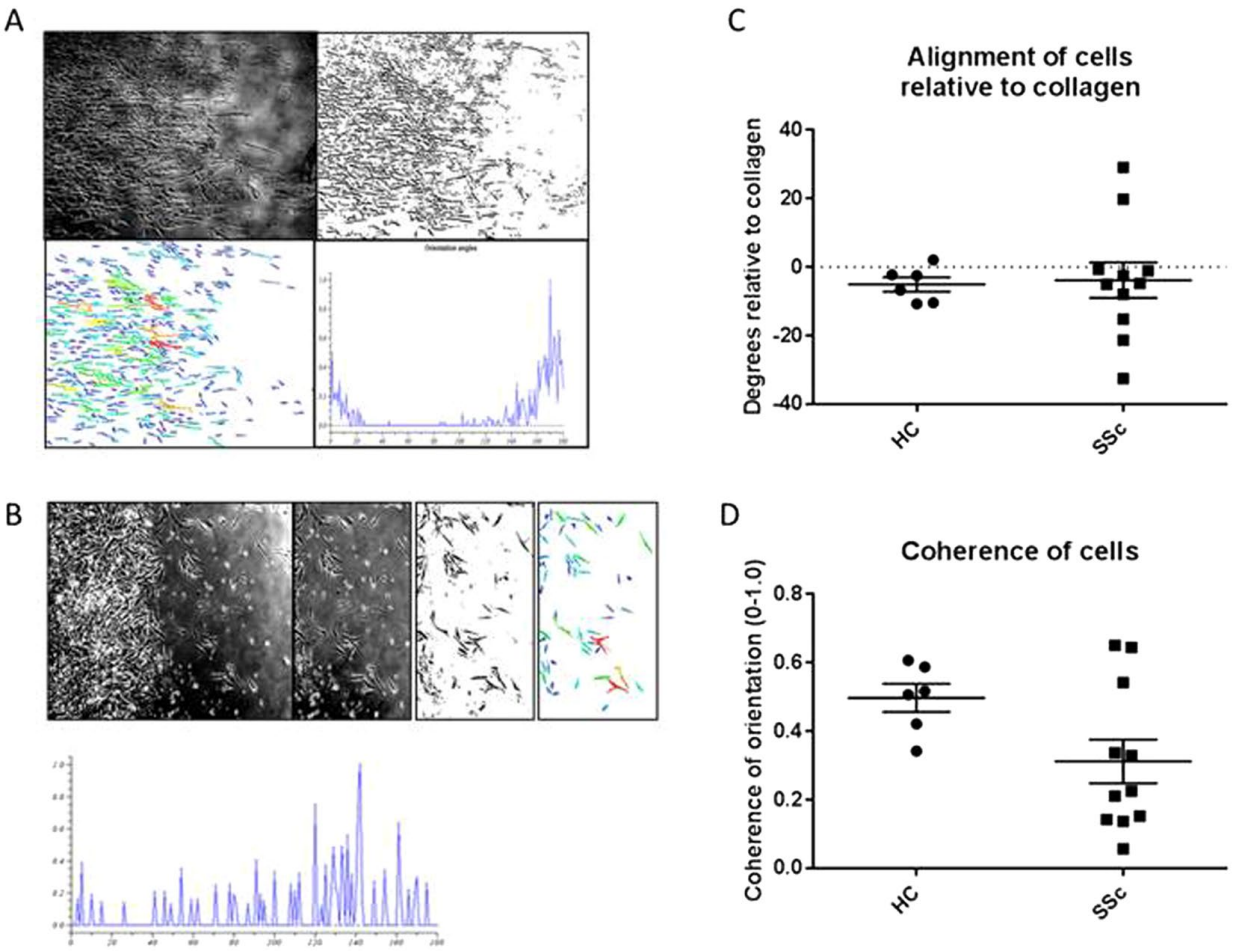
Altered orientation of SSc lung fibroblasts on aligned collagen. Control (**A**) and SSc (**B**) lung fibroblast lines were cultured as foci on unaligned and aligned collagen coated slides and allowed to migrate in DMEM with 10% FCS. Representative analysis steps are shown in sequence from left to right; original image, binary image derived from the selection of cells to analyse, and cluster analysis of the image to arrive at the profile of orientation angles, clusters around the 180 degree median is shown in the graphs. Control samples consistently aligned at 180° on linear collagen coated slides, whereas SSc lines diverged from the collagen alignment by up to 40° (**C**), and in general there was a lack of coherence of orientation in some but not all of the SSc cell lines (**D**).

Further experiments were next performed to compare the orientation of control and SSc lung fibroblast lines. It was apparent that the normal control lung fibroblasts deposited on the aligned collagen slide migrate with a similar orientation in parallel to the collagen fibers. SSc lung behaved similarly to the control fibroblasts, in that migration could only be consistently induced by culturing lung fibroblasts on aligned collagen with FCS. However, the orientation of the SSc lung fibroblasts differed from contol cells (Fig. 2).

### Altered orientation of SSc lung fibroblasts when cultured on aligned collagen

The orientation of the fibroblasts when migrating on aligned collagen-coated slides was also investigated. Control and SSc lung fibroblast lines (control n = 6 replicates, SSc n = 11 replicates) were applied as cellular foci to aligned collagen and migration followed over 24 hours. Cell alignment software (Orientation J) was used to study the orientation of the SSc and control cells. Whereas control fibroblasts aligned clustered at the 180° meridian in parallel to the aligned fibres, some SSc cell lines (5 out of 11 studied) failed to align with the collagen, although overall the area of migration was similar to controls (Fig. 2).

### Proteomic analysis of control and SSc fibroblast matrisomes

To explore whether or not SSc fibroblasts lay down different ECM proteins compared to control cells, we compared ECM preparations from coresponding cell cultures following 48 hours migration on aligned collagen (Table S2, Supplementary data). A higher number of ECM proteins was identified in preparations from SSc fibroblast compared to control fibroblast cultures. Amongst the SSc-specific proteins were the protease inhibitor TIMP1 and several involved in collagen modification, including three proline hydroxylases (LEPRE1/P3H1, LEPREL2/P3H3 and P4HA1) and two lysine hydroxylases (PLOD1 and PLOD3), while control-specific proteins included several other protease inhibitors (CSTB, SERPINB10/B2, and SERPINB6)^15^.

### The SCF/c-Kit pathway is involved in aligned lung fibroblast migration

One advantage of the aligned collagen migration assay is that it allows for the analysis of cells which have undergone migration, which can be lysed from the chip in sufficient numbers for profiling. In order to attempt a broad screening of the pro-migratory factors, cells were lysed following a 48 hour migration on aligned chips and the lysate subjected to Kinexus phosphorylation arrays, using matched non-migrating paired samples of lung fibroblasts cultured on the woven, non-migratory collagen as controls. A number of maximally enhanced phospho-proteins were identified as increased in the migrating cells (Table S1, Supplementary data). One surprising result was that the hematopoietic stem cell marker c-Kit was amongst the elevated phospho-proteins. This finding was evaluated further because although c-Kit is present on a subpopulation of lung stem cells as well as mast cells recruited into fibrotic lesions, it is not considered a fibroblast surface marker^16, 17^.

**Figure 3:**
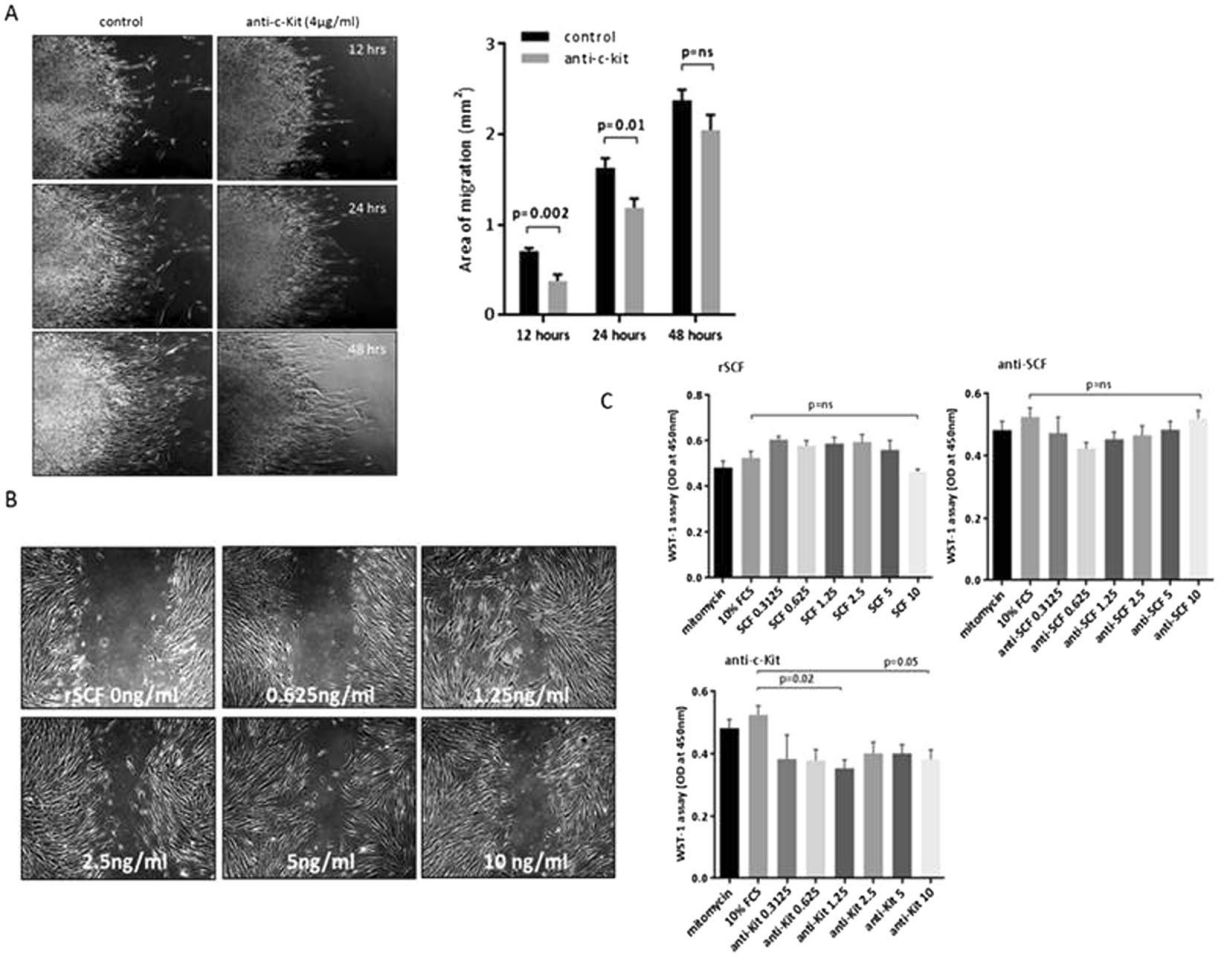
Role of SCF/c-Kit in regulating lung fibroblast migration and proliferation. 4 control lung fibroblast lines were applied to aligned collagen as cellular foci, and then stimulated to migrate using FCS 10%, with or without the addition of polyclonal anti c-Kit (4 µg/ml), and imaged at 12, 24 and 48 hours. After 12 hours the cell migration was significantly reduced (p = 0.002) in treated cells. Similarly, after 24 hours anti c-Kit decrease cell migration (p = 0.01) but there was no significant difference after 48 hours (**A**). Further experiments were performed with standard scratch migration assays. Here additional recombinant SCF (rSCF) enhanced scratch migration in control lung fibroblasts already stimulated by the presence of serum in the media. Maximal migration was observed when using 1.25 ng/ml of rSCF (p < 0.05 vs unstimulated cells) (**B**). Possible effects of the SCF/c-Kit axis on control lung fibroblast proliferation were investigated by adding rSCF as agonist or by blocking SCF/c-Kit with neutralising polyclonal anti-SCF or ant-c-Kit. The addition of rSCF (0–10 ng/ml) at the start of a 24 hour proliferation assay led to a non-significant increase in cell number, whereas addition of anti-SCF was associated with a trend to reduced cell number. Again not statistically significant. However neutralising anti-c-Kit blocked proliferation, significant at 2.5 µg/ml (neutralization dose (ND50) is typically 0.06–0.36 μg/mL in the presence of 20 ng/ml Recombinant Human SCF/c‑kit Ligand, R&D Systems #AF332) (**C**).

Polyclonal neutralising c-Kit antibody partially inhibited normal lung fibroblast migration on the aligned chips, and suppressed serum-induced proliferation (Fig. 3A). Stem cell factor (SCF), the ligand for c-Kit, is a pleiotropic growth factor regulating progenitor cell growth, and cellular recruitment to sites of injury^18, 19^. In further experiments using scratch wound migration assays, it was found that SCF enhances migration, working maximally when used at a concentration of 1.25 ng/ml (Fig. 3B). The role of the SCF/c-Kit axis in SSc fibrosis was explored further with emphasis on potential therapeutic strategies.

### Effect of the tyrosine kinase inhibitor imatinib on lung fibroblast migration

**Figure 4:**
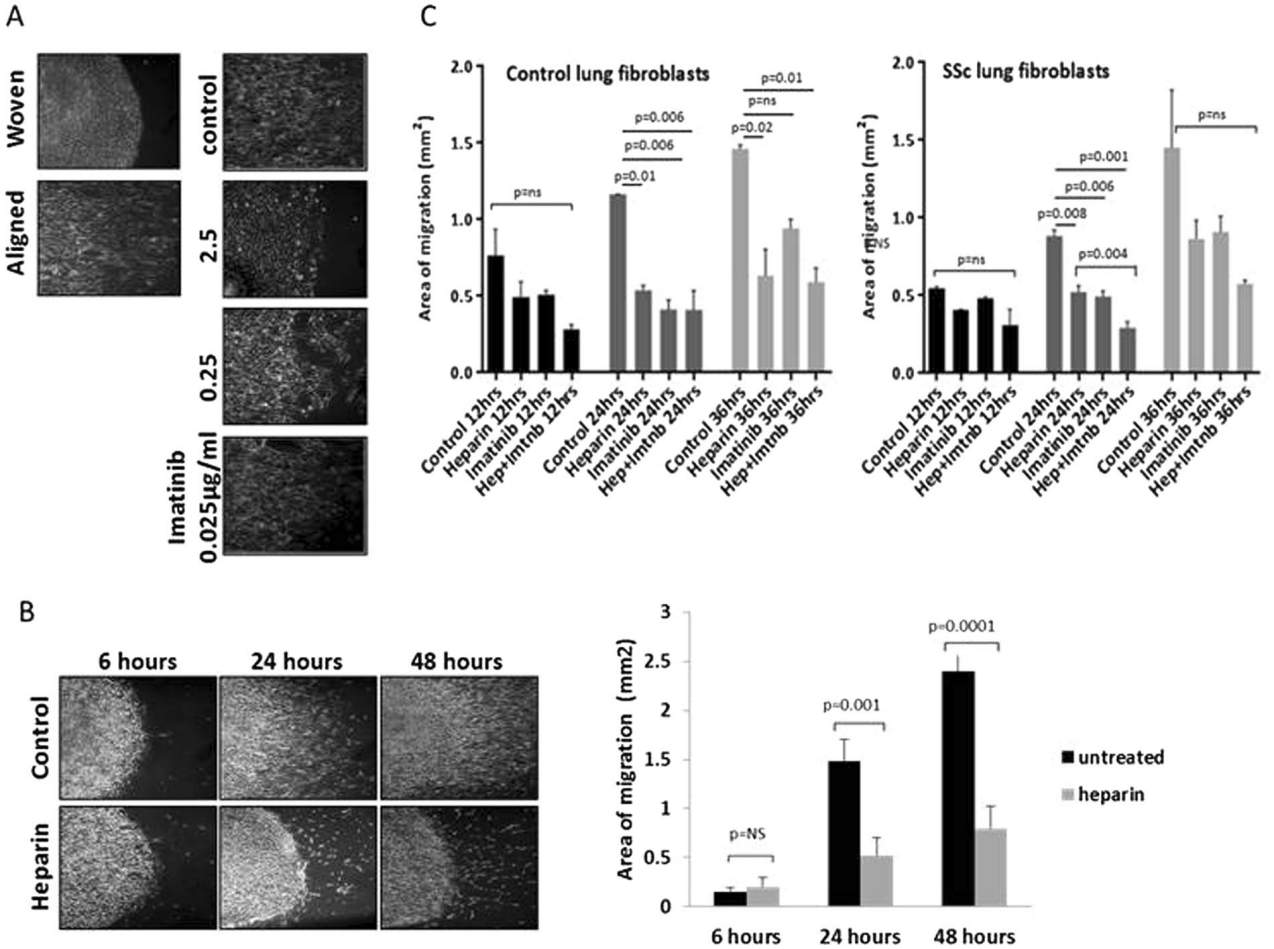
Effect of imatinib and heparin on lung fibroblast migration on aligned collagen. Initially the tyrosine kinase inhibitor imatinib, which blocks both PDGF receptor and c-Kit, was assessed for inhibition of aligned migration. Imatinib at 2.5 and 0.25 µg/ml inhibited aligned migration (**A**). Unfractionated heparin was also assessed as a possible inhibitor of migration. SSc lung fibroblasts were cultured on aligned collagen with or without the addition of heparin at 50 μg/mL to the media (DMEM plus 10% FCS). The migrating field of cells was quantified by ImageJ software, which outlined the migrating field of cells and then measured the area calibrated by the known dimensions of the 4 × axioscope fields. The addition of heparin inhibited cellular migration at 24 hours (p = 0.001) and 48 hours (p = 0.0001) (**B**). Combining heparin and imatinib in order to synergistically block growth factors as well as the receptor tyrosine kinases, was assessed. Combining both materials led to the lowest migration overall which was statistically significant for SSc cells (**C**). Control fibroblasts showed similar trends.

Imatinib has been used in clinical trials in SSc patients, with early trials showing encouraging, if somewhat equivocal results ( https://www.hss.edu/professional-conditions_new-drugs-to-treat-scleroderma-imatinib-mesylate-gleevec-trials.asp), including a negative phase II trial^20^. However, the activity of this tyrosine kinase inhibitor may be informative as to fibroblast migration and signalling. Potential blocking of migration on the aligned matrices was assessed using imatinib as an inhibitor of the PDGF receptor tyrosine kinase. The doses of imatinib tested were 0, 0.025, 0.25 and 2.5 µg/ml and the fibroblasts were cultured on the collagen slides for 24 hrs. Whereas the control cells on the unaligned collagen coated slide showed no directed migration, cells on the aligned collagen coated slides displayed moderate directed migration. In the portion of the study involving imatinib, the lowest dose (0.025 μg/ml) was similar to the no imatinib control, perhaps with slightly less migration toward the border of the slide. At the intermediate concentration (0.25 µg/mL), there was a clear reduction in cell migration and a seeming loss of orientation of the spread cells. At the 2.5 µg/mL level, there was a clear reduction in cell migration and a decrease in the orientation of the fibroblasts on the slide (Fig. 4A). Since imatinib inhibits the PDGF receptor, it is likely that it is blocking PDGF’s ability to stimulate migration.

### Effect of heparin on the aligned migration of lung fibroblasts

Heparin, a highly sulfated glycosaminoglycan obtained from various animal organs, has the highest negative charge density of any known biological molecule^21, 22^. It has been used clinically as an anticoagulant since the 1940s where heparin binds to plasma anti-thrombin with very high affinity, inhibiting a series of coagulation proteins including as thrombin and factor Xa. It also binds to numerous other proteins in a manner directly related to the bio-functionality of its structurally similar relative, heparan sulfate^23^.

The effect of heparin on the directed migration of SSc lung fibroblasts was tested using 10% FCS as stimulus. SSc fibroblasts were incubated on collagen-coated slides in the presence of 50 µg/mL heparin for 6, 24, and 48 hours. There was a significant decrease in migration by heparin treatment at 24 and 48 hours (Fig. 4B). These results show clearly that heparin inhibits the directed migration of SSc fibroblasts on aligned collagen when stimulated using 10% FCS. Based on the known activities of heparin, it may bind to growth factors and inhibit the activation of the PDGF receptor which utilize cell surface heparin sulfate as a co-receptor to stabilize growth factor-receptor interactions^24^.

It is likely that the attachment and alignment of the lung fibroblasts involves integrin receptors on the cell surface, with continual internalisation and recycling of cell surface integrins to the leading edge of the migrating cells. Chloroquine accumulates in lysosomes and is a known inhibitor of integrin recycling^25^. Chloroquine at 100 µM fully inhibited and at 10 µM, partially inhibited the aligned fibroblast migration, suggesting that integrins are involved and necessary for the migration by fibroblasts (data not shown).

### Synergy between imatinib and heparin as inhibitors of migration

We tested whether combining heparin, which binds to and inhibits PDGF activity in the extracellular environment, and imatinib, which blocks PDGF receptor (PDGFR) signal transduction, would lead to enhanced inhibition of migration. The impact of combination treatment using heparin plus imatinib on the directed migration of healthy and SSc lung fibroblasts was tested using 10% FCS as the stimulus. Control and SSc lung fibroblasts were incubated on aligned collagen-coated slides and imaged at 12, 24, and 36 hours. Predetermined optimum concentrations of heparin (50 µg/mL) or imatinib (2.5 µg/mL), or both factors were added to some of the cultures. There was a clear inhibitory effect of heparin on migration which reached statistical significance at the 24 and 36 hour time points in control lung fibroblasts, and at 24 hours in SSc lung fibroblast. Imatinib, as expected based on the experiments described above, suppressed migration reaching statistical significance at 24 hours in both control and SSc fibroblasts. The heparin/imatinib combination was associated with a trend towards lower migration then the single agent at all time-points, significant for SSc cells at 24 hours (Fig. 4C). Thus, the combination of 50 µg/mL of heparin and 2.5 µg/mL of imatinib shows synergy leading to greater inhibition of SSc lung fibroblast migration on aligned collagen coated slides.

### Effect of heparin and imatinib on scratch wound migration in SSc and control lung fibroblasts

In an attempt to validate these results, these inhibitors were studied in a standard scratch wound assay. However, no consistent effect was observed compared to that on the aligned collagen coated slides, implying a distinction in the mechanism of migration on the aligned coated slides versus the standard scratch migration assay carried out on plastic (data not shown).

### Assessing the activity of SCF and c-Kit in SSc and control lung fibroblasts

**Figure 5:**
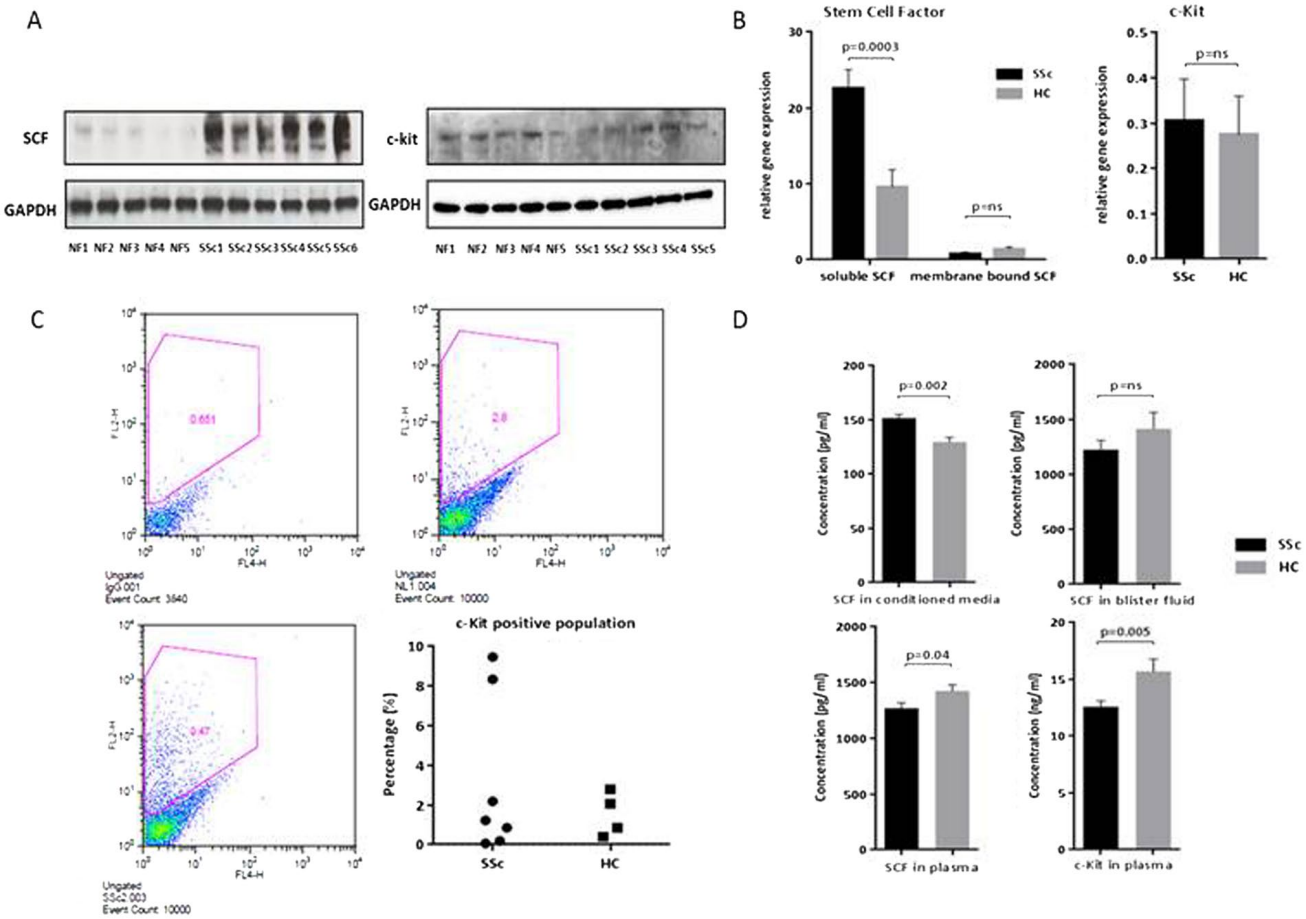
Activity of the SCF/c-Kit pathway in SSc cells and biologic samples. SSc and control lung fibroblasts both n = 5–6, were cultured. Western blot showed that SCF protein was increased in SSc cells compared to the controls. Uncropped films in supplementary data (**A**). qPCR revealed increased full length (soluble) splice variant of SCF in SSc cells, whereas the membrane bound isoform was present at lower and equivalent levels (**B**). C-Kit was expressed at low and similar levels in both SSc and control cells. FACS analysis of SSc and control lung fibroblasts revealed a c-Kit positive subpopulation (**C**). Representative IgG control shown upper left, healthy control lung fibroblasts upper right, SSc lung fibroblasts lower left. A small c-Kit positive sub-population were identified in both SSc samples as well as the control samples (mean for SSc samples 4.21%, mean for control samples 1.86% positive c-Kit cells). In addition, analysis of stem cell factor and c-Kit levels in fibroblast conditioned media, blister fluid and plasma was performed using ELISA. Measurement of SCF revealed a significant difference (p = 0.002) between SSc and HC in conditioned media but no significant difference in dermal blister fluid. Soluble c-Kit however was undetectable in both media and blister fluid (data not shown). In plasma, the healthy control group showed higher levels of both SCF (p = 0.04) and c-Kit (p = 0.005) (**D**).

Because of results showing involvement of c-Kit in lung fibroblast migration, further analysis of the expression levels of c-Kit and SCF in SSc and control cells was performed. Since SCF is known to be present as a soluble form due to cleavage at exon 6 by MMPs or an alternative splice variant in which the encoded protein lacks the protelytic cleavage site and therefore remains membrane bound, both isoforms were assayed. In SSc fibroblasts, both the protein levels for SCF and the mRNA for the soluble form of SCF were increased, indicating a possible role in paracrine signaling in these cells. C-Kit was transcribed and present at a low level overall, equal in SSc and control samples. FACS analysis revealed that the c-Kit was expressed by a minority subpopulation of cells (Fig. 5C).

Furthermore, the activity of extracellular c-Kit and soluble SCF were assayed by ELISA of tissue fluid, plasma, and conditioned media (Fig. 5D). However, there were only small differences between SSc and control levels suggesting that this pathway is not highly induced systemically, or that any activity is by local paracrine signalling.

**Figure 6:**
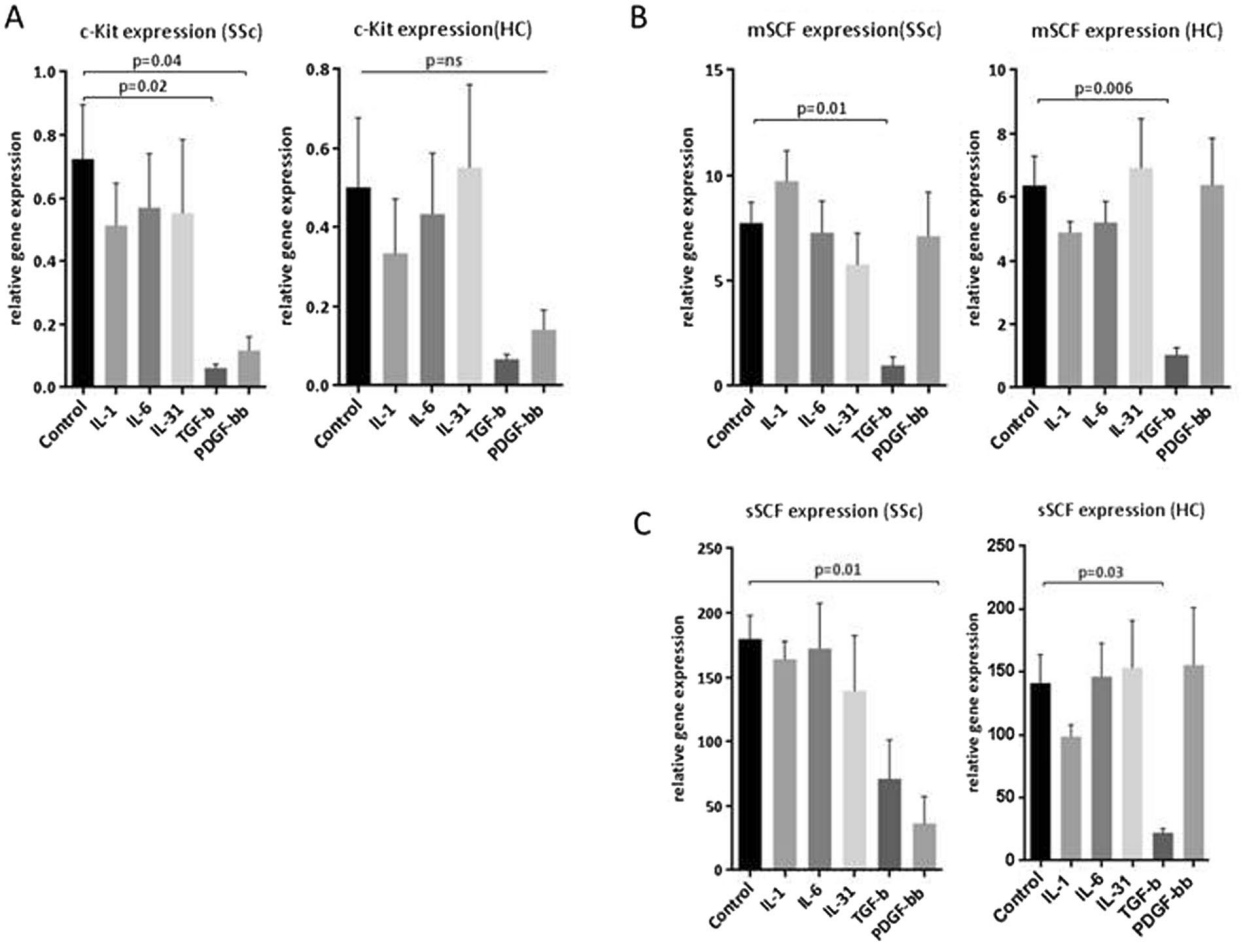
Regulation of SCF/c-Kit in lung fibroblasts by candidate cytokines and growth factors. SSc and control lung fibroblasts (n = 4 cell lines for both) were cultured in 6 well plates, switched overnight to serum free conditons, and then cultured for a further 24 hours with or withut candidate cyctokines and growth factors as shown. Cells were lysed and SCF/c-Kit assayed by qPCR. Gene expression of c-Kit was unaltered by inflammatory cytokines but greatly reduced by TGFβ and PDGF-BB, significant in SSc cells (**A**). Intriguingly, TGFβ, considered an important agonist in fibrotic disorders, also greatly inhibited both isoforms of SCF, whereas PDGF led to suppression of full length SCF in SSc cells only (**B**,**C**).

The regulation of the SCF/c-Kit axis was studied using candidate growth factors to reproduce inflammatory or pro-fibrotic environment present in the SSc disease lung tissue. In general, inflammatory cytokines did not influence the activity of SCF/c-Kit as assessed by qPCR. Interestingly, addition of the known pro-fibrotic growth factor TGFβ, considered a key driver of SSc fibrosis^26^, greatly reduced SCF and c-Kit expression (Fig. 6). It is possible that TGFβ is inducing commitment of the c-Kit positive population of cells. PDGF-BB, which is also a known inducer of lineage commitment in progenitor cells, also led to loss of c-Kit (Fig. 6A).

## Discussion

Pathological fibroblast migration is a relatively unstudied process in SSc, and to our knowledge, this is the first study attempting to model mechanisms underlying the spread of the fibrotic lung lesions. In the current study, no fibroblast migration occurred under low serum without added PDGF. However, migration took place when lung fibroblasts were cultured on aligned collagen following stimulation with serum or PDGF. Migration rates did not differ between SSc and control fibroblasts. PDGF has previously been shown to govern fibroblast migration, attracting fibroblasts to sites of injury^27, 28^. High levels of PDGF have been demonstrated in lesional fluid from SSc patients^13^. PDGF provides directionality and enhancement to fibroblast motility when cells are cultured on collagen^29^. An alternative explanation for enhanced migration of SSc fibroblasts in fibrotic tissue would be a higher concentration of the inducing factor PDGF.

Migration was not induced on the woven, randomly orientated collagen substrates. It is possible that an underlying molecular mechanism constrains fibroblast migration once cells have migrated into complex fibrotic tissues where they encounter collagen fibrils which, are randomly orientated, but permits migration through healthy tissue along aligned collagen. In keeping with this idea, detailed 3D reconstruction of pathologic lung fibrosis tissue has suggested that the fibrotic foci are linked by a continuum of aligned extracellular matrix which interconnects the fibrotic cellular sites along a reticulum network^30^ and that fibroblastic foci exhibit markers of migration and invasiveness^31^. Fibroblasts interact with the collagen matrices via cell surface integrins which bind to the collagen fibres and induce intracellular changes including protein docking and phosphorylation events which enhance motility^32^. Fibroblasts on aligned collagen may induce multiple sites of integrin adhesion as well as intracellular integrin modification, necessary for migration dependent on PDGF.

The abnormal alignment of some SSc lung fibroblast cell lines observed in the current study raises interesting questions. SSc fibroblasts have altered cell surface markers compared to control cells, including integrins which are important for the cell-ECM interactions^6, 7^. For example, previous studies indicate that SSc dermal fibroblasts have reduced levels of expression of α1β1 and α2β1 integrins and increased levels of αvβ5^6, 7^ and although these studies focused mainly on the synthetic function of fibroblasts, it may be that altered interaction also results in poor alignment. Integrin-ECM interaction is crucial for the development of polarity in fibroblasts as well as inducing cystoskeletal changes^33^. Alteration in these cell surface markers in disease fibroblasts may impair cell-ECM interaction, with resulting inability to align properly. Moreover, we have shown that the addition of chloroquine, which is an inhibitor of β1 integrin membrane cycling^25^, prevented aligned fibroblast migration.

Interestingly, analysis of the proteins secreted indicated major differences between the proteins secreted into the ECM by SSc and control lung fibroblasts. SSc lung fibroblasts were found to add collagens I and III to the ECM as well as prolyl and lysyl hydroxylases, which are involved in cross linking of the collagen leading to the triple helical form^27, 28^. These changes may stabilise pathologic ECM making it resistant to degradation by MMPs and other proteases, and may alter the way the disease fibroblasts interact with the ECM. Healthy control fibroblasts exhibited a different secretome, indicating that MMPs and Serpin inhibitors were released into the ECM by these cells, as well as transglutaminase I, a further cross linking enzyme not seen in the SSc secreted proteins. These changes could underlie differences between reparative and fibrotic ECM, or explain altered fibroblast-ECM interaction in fibrotic tissues.

Similarly to PDGF, SCF has a role in promoting the migration of lung fibroblasts. We observed that the receptor for SCF, c-Kit, was expressed by a subpopulation of the cultured cells, consistent with the known heterogeneity of primary fibroblast cultures^34^. C-Kit positive stem cells have been identified in lung tissue, present in the distal airways, and capable of the regeneration of epithelial structures^16^.

Increased SCF in SSc dermal fibroblasts has previously been demonstrated, highlighting its role in mast cell recruitment and fibroblast-mast cell cross talk in the disease^35^. We therefore chose to assess the SCF/c-Kit axis in lung fibroblasts. Quantitative PCR revealed that the expression of the full length soluble SCF was more than 2-fold higher in disease fibroblasts compared to controls. In contrast, there was no difference in gene expression of the membrane bound isoform between the two groups. Furthermore, neutralization of SCF’s effects by anti-c-Kit or through pharmacologic inhibitors, reduced proliferation and migration of lung fibroblasts both from healthy donors and from SSc patients. Overall, the data suggests that activated fibroblasts express and secrete SCF which acts in a paracrine fashion on a local c-Kit positive subpopulation, enhancing migration and proliferation in the lesions.

In contrast to previous work^36^, we did not confirm systemic elevation of plasma SCF. However, conditioned media from cultured fibroblasts was characterised by elevated soluble SCF levels in SSc patients compared to the controls Since earlier studies have also shown that SCF expression was markedly higher in active skin lesions in SSc (oedematous phase) compared to inactive (atrophic phase) lesions, it is likely that this pathway contributes to the overall disease process, through paracrine signalling as well as mast cell recruitment into the lesions.

TGFβ is a key pro-fibrotic factor implicated in SSc and a target for therapies^26^, and we therefore investigated possible cross-talk between TGFβ and the SCF/c-Kit pathway. However, contrary to expectations, addition of TGFβ shut down the SCF/c-Kit axis, indicating that these are distinct mechanisms. The findings are consistent with current models of scleroderma, as a complex disorder in which multiple signalling pathways, growth factors, cytokines and pathogenic cellular events all contribute to a pro-fibrotic state resistant to therapies. It is possible that TGFβ induces commitment of the c-Kit positive progenitor cells, causing a switch from regeneration to fibrosis in disease tissue.

Preliminary studies also showed that individual agents as well as combinations of potential therapeutics can be assessed by their effect on migration, based on the hypothesis that promising therapeutics can be detected by their activity as inhibitors of fibroblast migration. Accordingly, an experiment was designed to determine if imatinib inhibits the migration of lung fibroblasts on aligned collagen. Similarly, another experiment was conducted using aligned collagen slides with and without heparin, which binds PDGF as well as SCF and other fibroblast-related growth factors in the extracellular environment. We concluded that both imatinib and heparin significantly inhibit the ability of both SSc and control fibroblasts to migrate on aligned collagen coated slides. The combination of these two agents demonstrated synergy, with an enhanced response compared to the use of single agents in SSc fibroblasts. These results suggest that combining therapies that deplete the agonist growth factors, with those targeting the receptor tyrosine kinases could more completely arrest fibroblast invasion in pathological states.

At the time that these studies were underway, a multicentre clinical trial of imatinib as therapy for SSc skin involvement was carried out, ultimately with a negative outcome as well as dose-limiting toxicity^37^. However, in a recent open label study of prolonged low dose imatinib in SSc related lung fibrosis unresponsive to cyclophosphamide, stability or improvement of lung function was observed in 12 of 26 patients^8^.

Taken together these results show that, although the SSc-derived lung fibroblasts do not behave extraordinarily in terms of baseline or induced extent of migration, the elevated ligands PDGF and SCF in the SSc fibrotic lesion milieu may induce an amplified migratory response. Moreover, this SSc fibroblast response may be susceptible to down-regulation by targeting the PDGF receptor as well as c-Kit, with tyrosine kinase inhibitors, and heparin used separately or in combination. Our findings support a possible pilot evaluation of combination therapy.

## Methods

### Aligned collagen chips for fibroblast migration studies

Glass slides were coated with bovine collagen fibres by an application process developed at Fibralign, Union City, CA (www.fibralignbio.com), obtainable from Advanced Biomatrix, San Diego CA (www.advancedbiomatrix.com). This includes a woven dermis like pattern and aligned collagen fibres. Briefly, collagen coated slides were equilibrated in phosphate buffered saline for 5 minutes at room temperature, then washed in deionised water for 10 seconds prior to transfer into 70% ethanol for 1 hour to sterilise, then allowed to air dry. Subsequently, the chips were equilibrated in DMEM culture medium at room temperature for 1 hour, and then allowed to dry again for 15 minutes prior to use. SSc and control dermal and lung fibroblasts were cultured from explants of tissue biopsies obtained at skin punch biopsy or thorascopic biopsy, respectively, and studied at low passage number (3–5).

Near confluent cultures were detached using trypsin-EDTA and neutralised by adding an equal volume of Dulbecco’s Modified Eagle Media (DMEM) supplemented with 10% fetal calf serum (FCS). Cells were centrifuged at 1000 rpm for 5 minutes at 20 °C, then re-suspended in DMEM and counted to give a final cell density of 4000 cells per microliter. 3 µl cellular foci containing a total of 12,000 cells were added to each chip. 100 ul of DMEM culture media was added to wells in order to prevent evaporation of the media from the cellular foci and incubated for 1 hour. Finally, 4 ml of 37 °C DMEM with or without FCS was added and cells cultured further with or without addition of agonists or inhibitors.

Migration was quantified by imaging at 0 hour, 12 hours and 24 hours with Axioscope. Image analysis software (ImageJ, NIH) which outlined and measured the area of the migrating mass of cells, normalised to the area of the standard collagen coated tissue culture slide. In addition, Cell Align software was used to quantify orientation of the cells relative to the aligned collagen fibres.

### Matrisome analysis

To compare extracellular matrix proteins produced by SSc and control fibroblast, cultured fibroblasts were detached after 48 hours of migration on aligned collagen, using EDTA. The remaining extracellular matrix was extracted into lysis buffer prior to fractionation using SDS-PAGE, reduction and alkylation, trypsin digestion, HPLC and mass spectroscopy essentially as described^38^, but employing an Orbitrap Velos platform (Thermo). Raw data files were analysed using MaxQuant (v1.5.5.1) and the human canonical & isoform sequence database (Uniprot, downloaded 19^th^ July 2016). ECM proteins were identified using a human matrisome database^39^.

### Phospho-protein and collagen matrix profiling

Phosphorylated proteins were assessed in non-migrating and migrating conditions. Control and scleroderma lung fibroblasts were cultured as 40 × 12,000 cell foci on large format aligned and woven collagen slides, and after 48 hours migrating cells and non-migrating controls were lysed and then subject to Kinexus phosphorylation arrays to identify phosphor-proteins elevated during migration.

### Assays for c-Kit and SCF in tissue samples, body fluids, and cell culture

Soluble forms of SCF and c-Kit in plasma, and dermal blister fluid (for method of collection see ref. 13) of SSc patients and controls, as well as conditioned media from cultured cells were assayed by ELISA (R&D). In addition, cell lysates were subject to SDS PAGE Western blots and probed with polyclonal anti-SCF and anti-c-Kit antibodies (R&D) and developed against film with ECL detection substrate. RNA extracted from cultured lung fibroblasts from SSc patients and controls was purified by RNeasy mini kit (Qiagen) and assayed by qPCR for SCF full length (soluble form), SCF splice variant (membrane associated) as well as c-Kit. Candidate cytokines and growth factors including IL-1β (25 ng/ml), IL-6 (10 ng/ml), IL-31 (50 ng/ml), TGF-b (4 ng/ml) and PDGF-bb (5 ng/ml), were used to study the regulation of the SCF/c-Kit axis.

### Flow cytometry and FACS sorting

A c-Kit positive subpopulation was sought using early passage (1–3) lung fibroblasts from SSc patients and controls. Briefly, the fibroblasts were detached using EDTA, re-suspended in FACS buffer (2% FCS and 5 mM EDTA in PBS), counted and kept on ice. The cells were labelled with conjugated PE Mouse anti-human CD117 (BD Biosciences) and incubated in the dark for 30 minutes at 4 °C. The proportion of cells expressing c-Kit was determined using BD LSRFortessa.

## Electronic supplementary material

Supplementary Information
