# Supplementary material for: Use of Patterned Collagen Coated Slides to Study Normal and Scleroderma Lung Fibroblast Migration
Source: Sci Rep. 2017 Jun 1;7:2628. doi: 10.1038/s41598-017-02621-3 (PMC5453993; doi:10.1038/s41598-017-02621-3)
Supplement: Supplementary file 1 — Supplementary Information [file 41598_2017_2621_MOESM1_ESM.pdf]

Supplementary Data

Use of Patterned Collagen Coated Slides to Study Normal and Scleroderma Lung Fibroblast Migration

Bahja Ahmed Abdi[1*], Henry Lopez[2], Sarah Karrar[1], Elisabetta Renzoni[3], Athol Wells[3], Angela Tam[1], Oseme Etomi[1], J. Justin Hsuan[4], George R. Martin[2], Xu Shiwen[1], Christopher P Denton[1], David Abraham[1], Richard Stratton[1]

[1]Centre for Rheumatology and Connective Tissue Diseases, Royal Free Hospital Campus, University College Medical School, Rowland Hill Street, London, NW3 2PF, UK

[2]MuriGenics, Inc., 941 Railroad Avenue, Vallejo, CA, 94592, USA.

[3]Imperial College London, Royal Brompton Campus, Sydney Street,London SW3 6NP, UK

[4]Institute for Liver and Digestive Health, Royal Free Hospital Campus, University College Medical School, Rowland Hill Street, London, NW3 2PF, UK

Target Protein Name	Phospho Site (Human)	Full Target Protein Name	Globally Normalized Dermis-Like	Globally Normalized Linear	%Error Range Linear	Z-ratio (Linear, Dermis-Like)
Erk2	Pan-specific	Extracellular regulated protein-serine kinase 2 (p42 MAP kinase)	972	2826	10.88	2.03
CAMK2d	Pan-specific	Calcium/calmodulin-dependent protein-serine kinase 2 delta	2394	6789	8.43	1.98
CAMK2b	Pan-specific	Calcium/calmodulin-dependent protein-serine kinase 2 beta	2491	6515	14.21	1.83
Kit	Y936	Kit/Steel factor receptor-tyrosine kinase	1442	3701	3.66	1.79
CaMKK (CaMKK2)	Pan-specific	Calcium/calmodulin-dependent protein-serine kinase kinase	3373	8575	10.45	1.77
CASP7	Pan-specific	Caspase 7 (ICE-like apoptotic protease 3 (ICE-LAP3), Mch3)	2270	5724	2.16	1.76
CASP5	Pan-specific	Caspase 5 (ICH3 protease, ICE(rel)-III)	1219	3060	4.71	1.75
CaMK1d	Pan-specific	Calcium/calmodulin-dependent protein-serine kinase 1 delta	2016	5002	18.07	1.73
ErbB2 (HER2)	Pan-specific	ErbB2 (Neu) receptor-tyrosine kinase	1330	3287	14.68	1.72
CDK5	Pan-specific	Cyclin-dependent protein-serine kinase 5	3221	7838	2.34	1.69
CDK2	Pan-specific	Cyclin-dependent protein-serine kinase 2	1096	2614	19.84	1.65
Hsp90a/b	Pan-specific	Heat shock 90 kDa protein alpha/beta	5453	12997	2.33	1.65
CaMK4	Pan-specific	Calcium/calmodulin-dependent protein-serine kinase 4	3108	7236	3.65	1.60
CASP4	Pan-specific	Caspase 4 (ICH2 protease, ICE(rel)-II)	3704	8496	4.34	1.58
CDK4	Pan-specific	Cyclin-dependent protein-serine kinase 4	1440	3287	8.07	1.57
PKCa	Pan-specific	Protein-serine kinase C alpha	2886	1286	24.17	-1.54
PKA Cb	S339	cAMP-dependent protein-serine kinase catalytic subunit beta	2814	1251	2.46	-1.55
PKC	Pan-specific	Protein-serine kinase C alpha	3985	1764	1.30	-1.55
Vimentin	S34	Vimentin	4855	2113	2.85	-1.59
PTP1C	Pan-specific	Protein-tyrosine phosphatase 1C (SHP1, SHPTP1)	3535	1508	3.99	-1.62
PKA Ca/b	Pan-specific	cAMP-dependent protein-serine kinase catalytic subunit alpha/beta	3319	1376	26.59	-1.68
SOD (Cu/Zn)	Pan-specific	Superoxide dismutase 1	2877	1152	4.69	-1.74
VHR	Pan-specific	Dual specificity protein phosphatase 3	3899	1173	12.78	-2.29
PP2Cd	Pan-specific	Protein-serine phosphatase 2C - catalytic subunit - delta isoform	4181	1119	5.11	-2.51

Table S1. Phosphorylation profiling of aligned and non-aligned normal lung fibroblasts. Kinexus analysis showing phosphoproteins of increased (>1.5) or decreased (<1.5) abundance in migrating cells.

Supplementary Data

Group	Gene Name	Unique peptides	Sequence coverage [%]	Mol. weight [kDa]	Intensity	Protein
Control only	CILP	1	1.3	132.56	412810	cartilage intermediate layer protein
	CSTB	2	24.5	11.139	155530	cystatin B
	LGALS7	2	18.4	15.075	296800	lectin, galactoside-binding, soluble, 7
	MMP14	2	3.8	65.893	245920	matrix metallopeptidase 14
	S100A16	1	10.7	11.801	96256	S100 calcium binding protein A16
	SERPINB10; SERPINB2	2	7.5	28.463	360250	serpin peptidase inhibitor, clade B (ovalbumin), member 10; serpin peptidase inhibitor, clade B (ovalbumin), member 2
	SERPINB6	4	16.3	43.024	606800	serpin peptidase inhibitor, clade B (ovalbumin), member 6
	TGM1	1	4.3	40.628	96502	transglutaminase 1 (K polypeptide epidermal type I, protein-glutamine-gamma-glutamyltransferase)
SSc only	ANXA7	1	1.9	50.315	199460	annexin A7
	COL1A2	6	6.2	129.15	1857500	collagen, type I, alpha 2
	COL3A1	2	1.8	138.44	300140	collagen, type III, alpha 1
	DPT	1	5.5	24.005	135510	dermatopontin
	EDIL3	1	3.8	52.747	119100	EGF-like repeats and discoidin I-like domains 3
	F10	1	2.3	54.731	256440	coagulation factor X
	F13A1	1	11.6	15.951	172310	coagulation factor XIII, A1 polypeptide
	F2	4	11.8	70.505	1745500	coagulation factor II (thrombin)
	LAMB1	1	0.6	200.48	136580	laminin, beta 1
	LAMC1	1	0.9	177.6	195810	laminin, gamma 1
	LEPRE1	5	8.2	78.921	2170400	leucine proline-enriched proteoglycan (leprecan) 1
	LEPREL2	1	2	81.836	135110	leprecan-like 2
	P4HA1	1	24	61.049	2208600	prolyl 4-hydroxylase, alpha polypeptide I
	PLOD1	10	20.5	83.549	2915800	procollagen-lysine 1, 2-oxoglutarate 5-dioxygenase 1
	PLOD3	4	7.2	84.784	626960	procollagen-lysine, 2-oxoglutarate 5-dioxygenase 3
	PXDN	6	7.6	165.27	1873500	peroxidasin homolog
	S100A4	1	10.9	11.728	102590	S100 calcium binding protein A4
	SERPINA1	1	2.8	40.234	111420	serpin peptidase inhibitor, clade A (alpha-1 antiproteinase, antitrypsin), member 1
	SPARC	2	15.4	17.47	882830	secreted protein, acidic, cysteine-rich (osteonectin)
	THBS1	14	16.1	129.38	6713500	thrombospondin 1
	TIMP1	1	12.5	10.706	323820	TIMP metallopeptidase inhibitor 1

Table S2. Protein modification of extracellular matrices by SSc lung fibroblasts. SSc and control lung fibroblasts were cultured for 48 hours and then switched to serum-free conditions overnight before cells were detached using trypsin-EDTA. The remaining extracellular matrix was extracted, fractionated, digested and analysed using mass spectrometry and bioinformatics.

Supplementary Data

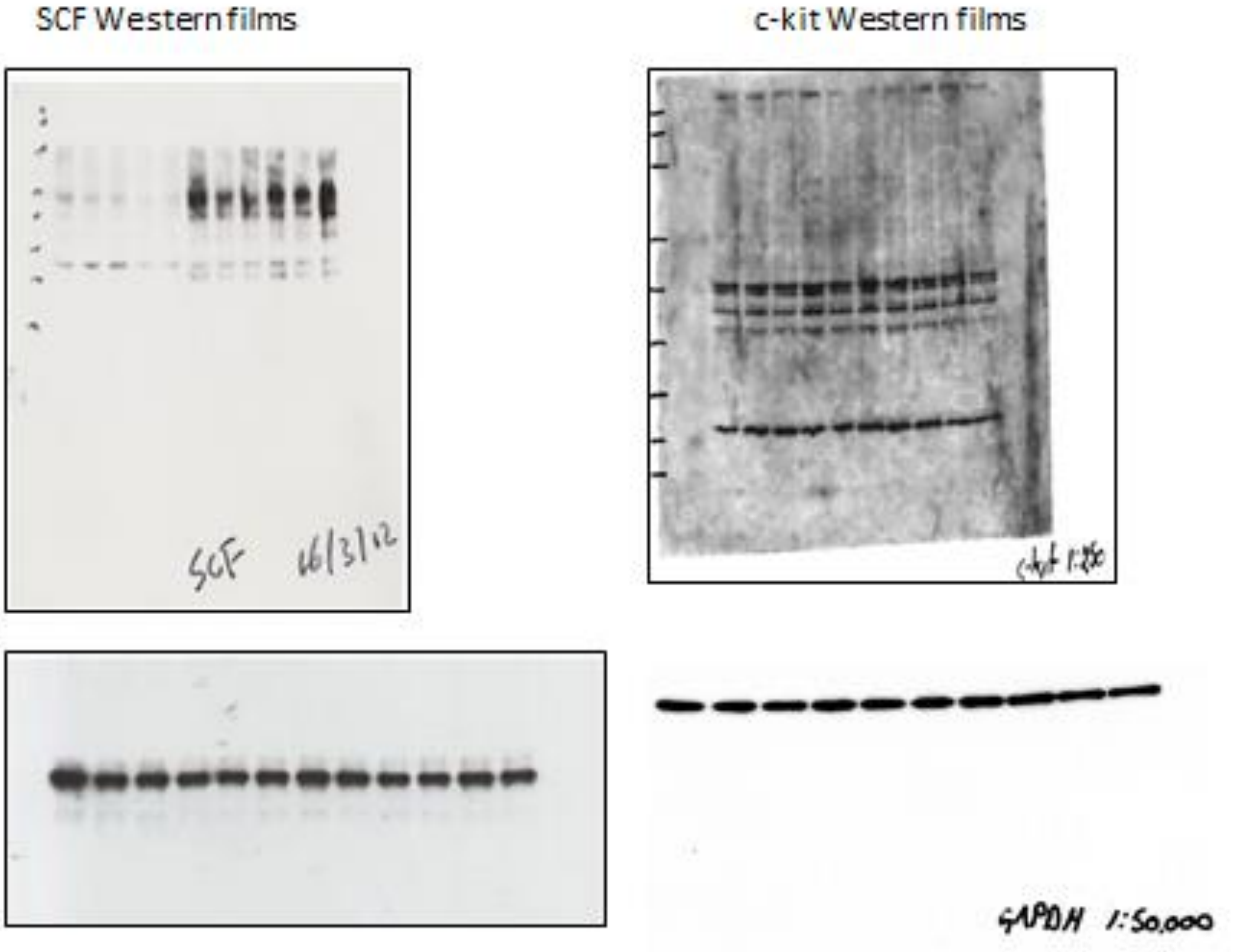

Figure S3. Scanned western blot films of stem cell factor, c-kit and their respective GAPDH. SSc and control lung fibroblasts both n=5-6, were cultured. Western blot showed that SCF protein was increased in SSc cells compared to the controls. There was no difference in c-kit protein amount between SSc and control fibroblasts.
